# Supplementary material for: Beeswax Alcohol (BWA, Raydel®) Improved Blood Oxidative Variables and Ameliorated Severe Damage of Zebrafish Kidneys, Testes, and Ovaries Impaired by 24-Week Consumption of a High-Cholesterol and High-Galactose Diet: A Comparative Analysis with Coenzyme Q10
Source: Pharmaceuticals (Basel). 2024 Dec 26;18(1):17. doi: 10.3390/ph18010017 (PMC11769329; doi:10.3390/ph18010017)
Supplement: Supplementary file 1 [file pharmaceuticals-18-00017-s001.zip › pharmaceuticals-3374639-supplementary.pdf]

Supplementary Material

Supplementary Method

Supplementary Method S1:

Materials: D-galactose (Cat#:G5388),N-ε-carboxymethyllysine (Cat#14580), coenzyme Q_{10} (Cat#303-98-0), 5-bromo-4-chloro-3-indolyl β D-galactopyranoside (Cat# B42525), oil red O (Cat#O0625), dihydroethidium (Cat#37291), acridine orange (Cat#A9231) were purchased from Sigma-Aldrich (St. L ouis, MO, USA). Beeswax alcohol (BWA) extracted at the National Center of Scientific Research (CNIC), Havana, Cuba, from the block beeswax of *Apis mellifera* (mainly mellifera lineage) was complimentary provided by the Raydel® Australia Pty. Ltd (Thornleigh, NSW, Australia). The extracted BWA (Cat#330020123) contained a typical mixture of six long-chain aliphatic alcohols (LCAA, C24-C34). A detailed specification of the BWA is enlisted as supplementary Table S1.

Supplementary Method S2:

Detection of reactive oxygen species and apoptosis in tissue: For dihydroethidium (DHE) [56] and acridine orange (AO) [57] fluorescent staining, the tissue section (7 μm thick) was covered with 250 μL of DHE (30 μM) and AO (30 μg/mL) solution. The section was incubated at room temperature in the dark after the 30 min-stained section was rinsed with water and observed under a fluorescent microscope for the detection of DHE and AO-stained area at the excitation and emission wavelength of 585/615 nm and 505/535nm, respectively.

Supplementary Table S1

RAYDEL
Health Confidence

Raydel Australia Pty Ltd
Level 1, building 1, 9-15 Chilvers Rd.
Thornleigh NSW 2120 Australia
Tel +61 2 9480 1300
Fax +61 2 9480 1399
A.B.N. 45 054 555 903
www.raydel.com.au
info@raydel.com.au

Certificate of Analysis

Product name: Beeswax alcohols

Batch #: 330020123

Date of Manufacture: 17/01/2023

Parameter	Results	Approved Limits
Aspect	Powder	Powder
Color	White bone	Off white to cream
**Identity and Purity***		
1-tetracosanol (C_{24})	6.12 %	6-15 %
1-hexacosanol (C_{26})	10.72 %	7-20 %
1-octacosanol (C_{28})	13.75 %	12-20 %
1-triacontanol (C_{30})	30.52 %	25-35 %
1-dotriacontanol (C_{32})	22.08 %	18-25 %
1-tetratriacontanol (C_{34})	2.95 %	≤ 7.5 %
**Total (Purity*)**	86.14 %	**≥ 85 %**
**Other quality specifications**		
**Melting temperature**	80.1-81.6 °C	78.0 – 85.0 °C
**Loss on drying**	0.60 %	≤ 1.0 %
**Heavy metals (Pb, Cd, Hg)**	<0.0000115 %	≤ 0.001 %
**Residual solvents**		
**Acetone**	≤ 0.03	≤ 0.03 g/kg
**Hexane**	≤ 0.005	≤ 0.005 g/kg
**Microbiological content ** **		
**Total Aerobic Microbial Count**	≤ 10	≤ 10^3 per g
**Yeast and mould**	≤ 10	≤ 10^2 per g
**Enterobacteria**	≤ 10	≤ 10^2 per g
***Staphylococcus aureus, Pseudomonas aeruginosa, Escherichia* coli, Candida albicans**	Absent	Absent in 1 g
Salmonella sp	Absent	Absent in 10 g
**Observations**		
**References:**	* Manufacturer GC validated method; purity expressed as the total of high molecular weight alcohols.	
	** BP	

Note about storage conditions: No special storage conditions are required. The substance has a shelf life of 5 years stored under ambient conditions of climatic Zones IV or II, as demonstrated in stability studies performed according to ICH guidelines.

Approved (_X_) Released (_X_) Rejected (___)

This COA is it reproduced from supplier's COA

Supplementary Table1: Certificate of analysis and composition of beeswax alcohol (BWA) used in the present study.

Supplementary Figure S1

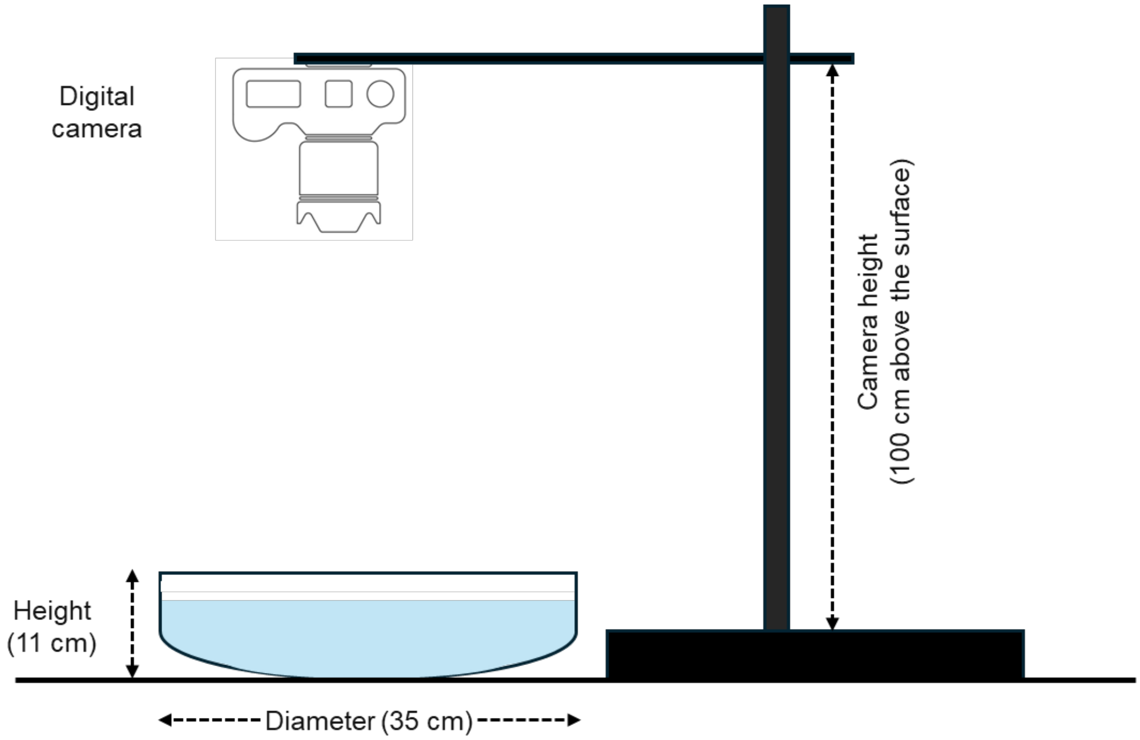

Supplementary Figure S1: Ray diagram of zebrafish swimming activity analysis experimental system.

References:

56. Owusu-Ansah, E.; Yavari, A.; Mandal, S.; Banerjee, U. Distinct mitochondrial retrograde signals control the G1-S cell cycle checkpoint. *Nat. Genet.* **2008**, *40*, 356–361.
57. Umali, J.; Hawkey-Noble, A.; French, C.R. Loss of foxc1 in zebrafish reduces optic nerve size and cell number in the retinal ganglion cell layer. *Vision Res.* **2019**, *156*, 66–72.
